# Creating reproducible pharmacogenomic analysis pipelines

**DOI:** 10.1038/s41597-019-0174-7

**Published:** 2019-09-03

**Authors:** Anthony Mammoliti, Petr Smirnov, Zhaleh Safikhani, Wail Ba-Alawi, Benjamin Haibe-Kains

**Affiliations:** 1 Princess Margaret Cancer Centre, University Health Network, Toronto, Ontario Canada; 2 Department of Medical Biophysics, University of Toronto, Toronto, Ontario Canada; 3 Department of Computer Science, University of Toronto, Toronto, Ontario Canada; 4 Ontario Institute of Cancer Research, Toronto, Ontario Canada; 5 Vector Institute for Artificial Intelligence, Toronto, Ontario Canada

**Keywords:** Cancer models, Data processing, Clinical pharmacology

## Abstract

The field of pharmacogenomics presents great challenges for researchers that are willing to make their studies reproducible and shareable. This is attributed to the generation of large volumes of high-throughput multimodal data, and the lack of standardized workflows that are robust, scalable, and flexible to perform large-scale analyses. To address this issue, we developed pharmacogenomic workflows in the Common Workflow Language to process two breast cancer datasets in a reproducible and transparent manner. Our pipelines combine both pharmacological and molecular profiles into a portable data object that can be used for future analyses in cancer research. Our data objects and workflows are shared on Harvard Dataverse and Code Ocean where they have been assigned a unique Digital Object Identifier, providing a level of data provenance and a persistent location to access and share our data with the community.

## Introduction

With the advances of high-throughput technologies in biomedicine, the volume of data has drastically increased in the last decade across scientific disciplines^1^. This influx of data has provided researchers with the ability to discover and utilize data of various types and structural characteristics that aid in carrying out leading-edge research. However, when heterogeneous and multimodal data types are produced in large quantities, the data become much more complex to process, making conventional computational processing methods inadequate and calling for new solutions^2,3^. These conventional methods encompass the use of scripting languages to process this data lacking (*i*) resource management capabilities (compute and memory); (*ii*) ability to aggregate data from multiple sources; (*iii*) support for modular processing; (*iv*) ability to handle unstructured data; and (*v*) ability to transform data to be used with other tools/algorithms^4,5^. Moreover, pipelines harnessing complicated methods for processing cancer pharmacogenomic data, which is data measuring the way a cancer’s genome affects its response to drug therapy (multiple gene-drug associations), may be difficult to reproduce^6,7^. These methods include the use of convoluted scripts that deploy multiple genomic tools and statistical methods/algorithms to compute drug response and identify molecular features^7,8^. Studying the effects of a drug on a single gene (single gene-drug association) or a few genes, is referred to as a pharmacogenetic analysis^6^. A challenge subsequently arises, as there becomes a plethora of pipelines for pharmacogenomic datasets that utilize different complex methods, which all aim to perform the same goal, but will yield different results^9^. These limitations hinder scalability and the use of pharmacogenomic data generated by drug screening facilities worldwide, to its full potential. There is therefore a need for the development of more sophisticated computational pipelines to address these issues^10^.

To address the issues of scalability, reproducibility and standardization with processing and analyzing pharmacogenomic datasets, we created open-source processing pipelines using the Common Workflow Language (CWL), a popular data workflow language in the data science and bioinformatics community^11^. We leveraged *PharmacoGx* within our pipelines, an R/Bioconductor package that provides computational approaches to simplify the processing and analysis of such large datasets^12^. We pushed our CWL pipelines to Code Ocean^13^, which process two large breast cancer pharmacogenomic datasets^14–17^ and create fully documented data objects shared through a persistent, unique digital object identifier (DOI) on Harvard Dataverse^18^. Our study demonstrates how existing computational tools and platforms can be used to standardize the processing of pharmacogenomic data in a transparent and reproducible way, and how these processing pipelines and resulting datasets can be shared with the scientific community.

## Pharmacogenomic Datasets

The first dataset is the Oregon Health and Science University (OHSU) breast cancer screen generated within Dr. Joe Gray’s laboratory (GRAY)^14,17,19^. The two most recent versions of the GRAY dataset were published in 2013 and 2017, where the latest update collectively includes 91 cell lines and 107 drugs, with 9,756 drug sensitivity experiments for 72 cell lines screened against 107 drugs, after our curations^14,17,19^. The dataset includes processed SNP (*n* = 77), exon array (*n* = 56), U133A expression (*n* = 51), RNA-seq (*n* = 54), RPPA (*n* = 49), and methylation (*n* = 55) profiles with the use of various technologies and processing methods (Table 1)^17,19^. Multiple cell lines were added to the GRAY molecular profile data after the 2013 release, but before the update to the drug response data in the 2017 release, resulting in our curation of 91 cell lines for both versions of the dataset^14,17,19^.

**Table 1:** Summary of cell line and drug curations, sensitivity experiments, and molecular profile processing for GRAY and UHNBreast datasets.

	GRAY 2013	GRAY 2017	UHN Breast 2017	UHN Breast 2019
Cell lines	91	91	83	85
Drugs	89	107	4	8
Experiments	9413	9756	52	689
Molecular data and processing	RNA-seq (ALEXA-Seq, TopHat, HTSeq)		RNA-seq (STAR, Cufflinks)	
	CNV (aroma.affymetrix, CNTools, DNACopy)		CNA (Illumina GenomeStudio, CNTools, DNACopy)	
	Methylation (Illumina GenomeStudio)		miRNA (sva, ComBat)	
	RPPA (normalization methods from MD Anderson)		RPPA (normalization methods from MD Anderson)	
	RNA (RMA, MicroArraySuite, aroma)			

The second dataset is the University Health Network (UHN) breast cancer screen (UHNBreast) with molecular and pharmacological profiles released in 2016^16^ and 2017^15^, respectively. The dataset includes processed SNP (*n* = 79), RNA-seq (*n* = 82), RPPA (*n* = 79), and miRNA (*n* = 82) (Table 1)^16^. We provide the most recent update to UHNBreast with four new drugs (trastuzumab, olaparib, BYL719, and UNC0642), for a total of 85 cell lines, 8 drugs, and 689 drug sensitivity experiments, after our curations, where 56 cell lines were screened against 8 drugs^15,16^.

**Fig. 1:**
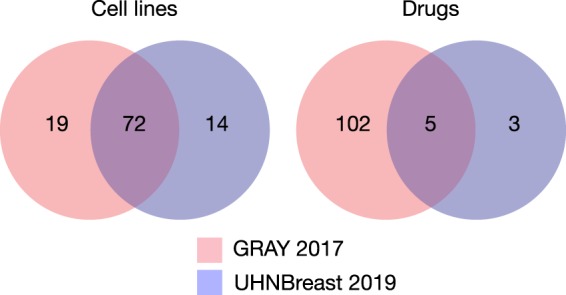
Convergence of drugs and cell lines between GRAY (2017) and UHN Breast (2019) after curation through our CWL pipelines.

The convergence of the 2017 update of GRAY and our 2019 update to UHNBreast yield an intersection of 72 cell lines and 5 drugs after curation through our pipelines (Fig. 1).

## Reproducible and Transparent Processing of Data

**Fig. 2:**
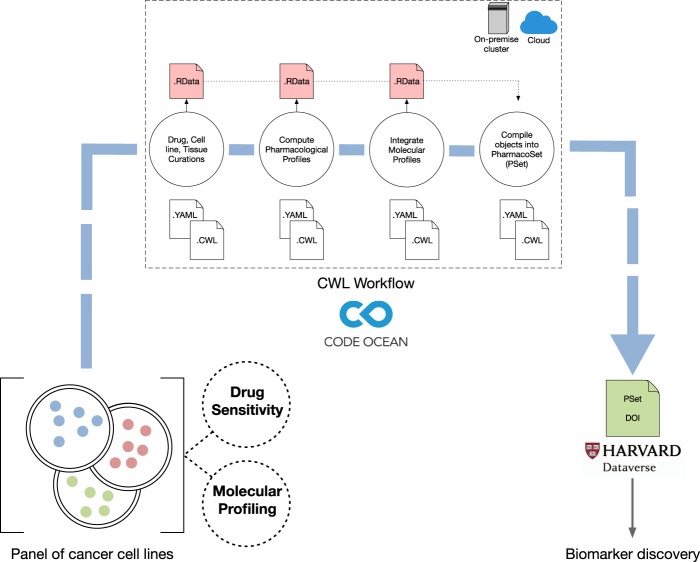
Breast cancer PharmacoSet (PSet) generation and DOI assignment through execution of a reproducible *PharmacoGx* CWL workflow.

Due to the scale and complexity of data that are produced through high-throughput platforms, the data processing and analysis pipelines should possess a robust and flexible infrastructure^4,5^. It is therefore important for pipelines to support interoperability, such as where different tools can be allocated to different data^20^. However, pipelines that are interoperable by consisting of multiple components/stages are difficult to reproduce^21^. To solve this issue, we developed our *PharmacoGx* pipelines in CWL, which allowed us to standardize the way we executed our multi stage processing and analysis of both breast cancer datasets in a reproducible and transparent manner (Fig. 2) (see Methods)^11^. Importantly, *PharmacoGx* implements the PharmacoSet (PSet) class, allowing us to create shareable R objects integrating all aspects of pharmacogenomic datasets, from cell line and drug annotations to the molecular and pharmacological data^12^. Each CWL pipeline is allocated a specific subroutine that is required for PSet creation, which includes curating cell and drug annotations, computing drug response, and incorporating processed molecular profiles for a given dataset (Table 2)^11,12^. To accomplish this in a semi-automatic fashion, we incorporated each pipeline into a CWL workflow, where *PharmacoGx* computes each stage of a pipeline and assembles their corresponding outputs into a PSet. This workflow not only transparently indicates the pipelines that are being executed, but also ensures that each pipeline is executed in the same manner if replicated, enforcing reproducibility^11^. In addition, the support of interoperability through CWL can be highlighted, as each pipeline stage that generates a corresponding output interacts with subsequent stages, which can be further enforced through specifying file-specific ontologies. These pipeline interactions validate the integrity of each given output object and PSet generated to ensure that it can be used for secondary analyses^11,12^. Interoperability through shared ontologies is also supported by *PharmacoGx*, as our pipelines curate and assign unique identifiers to each cell line and drug compound in each dataset, where the identifiers are used in subsequent pipeline stages to verify that the data is correctly compiled^12^. Therefore, the unique identifiers not only validate PSets that are generated, but also maximize consistency across existing PSets. However, because every dataset requires a different way of transforming and processing the data, due to variability in the way the data were initially shared and structured for each study, GRAY and UHNBreast possess their own CWL pipelines and workflow to accommodate for the differences^14–17,19^. Because CWL is a standardized language, each pipeline must include input and output definitions, base commands, and requirements (e.g., resource, Docker)^11^. In addition, each CWL pipeline and workflow must be accompanied by a YAML (YAML Ain’t Markup Language) or JSON file, which consists of an object array that defines a class and path for each input in the respective pipelines. In order for our CWL pipelines to execute successfully, they must specify the following: hints (docker requirement to run *PharmacoGx*), inputs that declare a type and input binding position (Rscripts, annotation files, raw drug data, processed molecular data), outputs that declare a type and output binding (e.g, processed drug sensitivity R objects, PSets), and a base command (to run Rscript), in the specified CWL file^11,12^. Therefore, in order for our CWL workflows to be fully documentented and reproducible, each pipeline must be defined as an input and possess a successful runtime independently^11^. Having to explicitly specify these parameters required to run each pipeline, along with the inputs and outputs in CWL provides an added layer of transparency to the pipelines, as well as allowing users to have control over data provenance. One of the highlights of our CWL workflows is the computation of drug response data for both datasets, which include AAC (Area Above the drug-dose response Curve), IC_50_ (maximal drug concentration to achieve 50% cell growth inhibition), Hill-Slope (measurement of slope of a drug-dose response curve), E_inf_ (maximum theoretical inhibition), and EC_50_ (drug concentration for which 50% of maximum response is observed) (see Methods). Computed AAC was later utilized in a post-PSet analysis to determine the concordance between a gene-drug association in both datasets through calculating the concordance index (CI) between respective RNA-seq and the AAC data (see Methods)^14–17,19^. For GRAY, we computed AAC, IC_50_, Hill slope values, and included published GI_50_, GR_50_, GEC_50_, GR_max_, GR_inf_, h_GR_, and GR_AOC_, data^14,17^ (see Methods). For UHNBreast, recomputation of AAC, IC_50_, and Hill slope was also performed, along with E_inf_, and EC_50_^15^.

**Table 2:** CWL workflow pipelines and their respective data streams to produce a PharmacoSet (PSet) for GRAY and UHNBreast datasets.

CWL Pipeline	Pipeline Description	Input	Output
Cell line Curation	Curates cell lines	Cell line annotation	Curated cell lines
Tissue Curation	Curates tissues	Cell line annotation	Curated tissues
Drug Curation	Curates drugs	Drug annotation	Curated drugs
Cell line Info	Collects cell line metadata	Cell line metadata	Cell line metadata
Drug Sensitivity	Recomputes raw drug response data	Raw drug response data	Recomputed sensitivity
Drug Published	Collects published drug response data	Published drug response data	Published sensitivity
Molecular Profiles	Incorporates molecular data into ExpressionSets	Molecular profiles	ExpressionSets
getPSet	Creates PSet	All objects produced by each pipeline	PSet

## Tracking Data Provenance and Validating Pipeline Integrity

Tracking data provenance with CWL can be further enhanced through the use of the provenance flag (–provenance) when executing the PSet workflows^11^. Here, a Research Object is automatically generated, which is a directory that acts as a bundled container for all of the resources utilized and produced within our workflows, including metadata that annotates each resource^11,22^. Within this object is a “data” directory that contains each input file used in the workflow with a unique and fixed checksum^11^. We are given granular transparency across the entire workflow at every stage, as we are able to map each checksum to a respective input file and location in the “data” directory, including all of the Rscripts that were utilized within a pipeline, through a workflow metadata file that is generated. In addition to a checksum, each PSet is also assigned a Universally Unique Identifier (UUID), which provides an additional layer of provenance to accurately identify the PSet that was generated by the workflow^11,12^. Moreover, this is accompanied by a provenance metadata file, which provides users with the ability to use checksums and UUID’s to accurately identify when each file was called and generated along the entire execution of a workflow^11^. Therefore, a Research Object confirms the reproducibility of our CWL workflows and validates the PSet that was generated with a respective runtime by providing rich metadata that tracks data provenance at each stage of a workflow.

## Harnessing Docker to Create a Reproducible Runtime

*PharmacoGx* integrates seamlessly with CWL, as we leverage CWL’s Docker capabilities to containerize the package and run all of our pipelines in an isolated environment^11,12,21^. Docker is a tool that allows for *PharmacoGx* to be uniformly deployed with all software dependencies, in a containerized runtime environment where all of our computations are performed and PSets are produced^12,21^. The Docker container is invoked upon CWL workflow execution, where all the input files for a given pipeline become mounted into the container and all output files produced in the isolated environment are recovered into a local environment^11,23,24^. Another advantage of Docker is the ability of containers to utilize and share the hardware resources of the environment it is being run in^25^. Therefore, *PharmacoGx* deployment is not only consistent, but also portable across both cloud and high performance computing environments, as our Docker image is also publicly available through Docker Hub (https://hub.docker.com/r/bhklab/pharmacogxcwl)^24,25^. The ability to standardize the manner in which PSets are produced through CWL and develop an additional layer of abstraction for pipeline execution through Docker, allowed us to create and deploy reproducible and transparent pharmacogenomic pipelines that can be shared with the research community and replicated.

## Sharing of Data and Pipelines

In order for a study to be computationally reproducible, data and pipelines must be well documented, uniquely identified, and easily accessible in a persistent location to other researchers^26^. To accomplish this, we utilized the Harvard Dataverse to share our PSets for both breast cancer pharmacogenomic datasets, along with Code Ocean to share our CWL pharmacogenomic pipelines^13,18^. Harvard Dataverse is an online data repository for transparently preserving and sharing research data with other researchers^18^. By creating a container known as a “dataverse” within the platform, researchers are able to deposit their datasets and corresponding metadata, in an organized fashion and make them easily discoverable for others to download and share. Each dataset can be also assigned a unique DOI, which allows a dataset to possess a persistent location, as well as allow researchers to accurately identify and share a specific dataset of interest. In addition, subsequent updates (versions) to a dataset can be uploaded, with accompanying metadata that explains the update and its changes, providing a layer of data provenance to the research community.

We also transferred our reproducibility measures to the pipeline level, as we deposited and shared our CWL workflows through Code Ocean, a reproducibility platform that allows for researchers to upload, share, and run published and configured code^13^. Data is uploaded into a “capsule”, which provides a computational environment for others to run code in the capsule, without the need to manually execute it locally with the addition of installing any dependencies^13,27^. Moreover, code can also be assigned a persistent DOI, providing the ability to accurately share and retrieve pipelines, as well as verify the reproducibility of published results directly through the compute capsule. Because Code Ocean does not currently support running multi-container pipelines, and therefore our CWL workflows, we used the platform to host our workflows and raw data, provide execution instructions, and run a post-PSet analysis for biomarker discovery.

Our PSets can be found on Harvard Dataverse at the following 10.7910/DVN/BXIY5W ^28^. Our CWL workflows can be found on Code Ocean at the following 10.24433/CO.7378111.v3 ^29^.

## Utilization of PSets for Biomarker Discovery

**Fig. 3:**
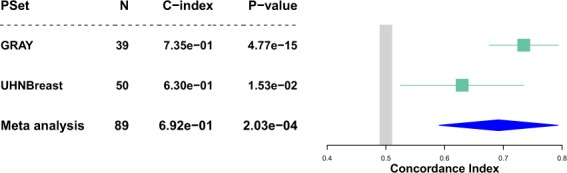
ERBB2 expression as a biomarker for lapatinib in GRAY 2017 and UHN Breast 2019. N: number of samples; C-index: concordance index calculated for respective PSet; P-value; p-value calculated for respective PSet. Meta analysis represents combined concordance index and p-value across PSets.

In order to demonstrate the utilization of our PSets for cancer research, we identified ERBB2 expression as a biomarker for lapatinib in both the GRAY 2017 and UHNBreast 2019 datasets (Fig. 3) (see Methods). To investigate this pharmacogenetic association^6^, we utilized processed RNA-seq expression and computed drug response (AAC) from each PSet^14–17,19^. We subsequently identified 39 cell lines from the GRAY PSet and 50 cell lines from the UHNBreast PSet that include both gene expression data for ERBB2 and drug response data for lapatinib and computed the strength of significance of this gene-drug association using the concordance index (CI). CI estimates the probability that random pairs of samples will be similarly ranked by two variables, in order to identify the agreement (concordance) between the two variables^30–32^. We found that ERBB2 expression was strongly predictive in both the GRAY and UHNBreast datasets (CI = 0.73, p-value = 4.8E-15 in GRAY and CI = 0.63, p-value = 0.015 in UHNBreast). This argues against the null hypothesis that ERBB2 expression is independent of lapatinib response. This analysis can be reproduced through our Code Ocean capsule^29^.

## Discussion

The utilization of CWL allows us to create and execute transparent and reproducible pharmacogenomic pipelines that can be validated and easily shared with the scientific community^11^. The standardized architecture of the language allows users to create language-agnostic pipelines and workflows that enforce strict parameter specifications to ensure execution is consistent. In addition, users are able to incorporate Docker into their runtimes, where data ingestion, analysis, and exportation all occur in an isolated container environment that promote repeatable execution^11,23,24^. Users are also able to track data provenance across the entire execution time by creating Research Objects in CWL, which validates each portion of data flow from input to output, through checksums and UUID’s^11,22^. Lastly, CWL pipelines and workflows are scalable and portable across many computing environments, such as the cloud, which gives users the ability to easily share their analyses and harness a plethora of various hardware resources to successfully execute their workloads that would not be possible with using on premise resources^11,24,25^. A common practice in pharmacogenomics is sharing study data as supplementary files through a journal, or through online sharing platforms/repositories such as Synapse and GitHub, which was the case for both the GRAY and UHNBreast datasets^14–17,19^. However, the challenge becomes assembling these data into a form that can be successfully analyzed and interpreted when shared. We were able to accomplish this in a reproducible manner by utilizing study data from a variety of sources and assembling it into a meaningful and useful form for cancer researchers, which are PSets, through CWL and *PharmacoGx*^11,12^. Therefore, our pipelines form the bridge between raw pharmacogenomic data and assembly in a transparent fashion. With our pipelines utilizing the versatile PharmacoSet class, many data types from other datasets can be easily encapsulated for PSet generation, such as methylation, chromatin accessibility (e.g., ATAC-seq), metabolomics, protein expression^33^, and radiation therapy response^34^, given that it follows the PharmacoSet data structure^12^. This provides many opportunities for researchers to process and analyze a plethora of data for their studies. However, our workflows do have limitations, including the inability to identify changes to pipelines, input data, and PSets, at the file level, when updates are pushed, and the files are taken into an environment outside of Harvard Dataverse and Code Ocean. However, with storing our data on Harvard Dataverse and pipelines on Code Ocean with rich metadata, users will be able to retrieve any updated files on both repositories and accurately identify the exact changes to each file. In addition, CWL Research Objects provide checksums and UUID’s only after a runtime is complete, which are bound to the file name and not persistently attached to a file for use in subsequent workflow runs^11^. Thus, if an input file is updated and re-utilized in a workflow, we must manually keep track of all checksums and UUID’s that were assigned to it by CWL over time. In the future, we hope to increase transparency and reproducibility by automating these pharmacogenomic pipelines in a manner that keeps track of all input and output data at the file level through the use of automatically generated unique identifiers that are persistent. Moreover, we hope to provide users with an interface that provides options for processing drug sensitivity and molecular profiles in a generated PSet.

## Methods

### Computation of drug response data

Our CWL pipelines process raw pharmacological data of the GRAY and UHNBreast datasets^14,15,17^. This encompasses the computation of AAC, IC_50_, Hill-Slope, E_inf_, and EC_50_. With regard to the sensitivity metrics, drug potency and efficacy is a measure of AAC, potency is a measure of IC_50_ and EC_50_, while E_inf_ is a measure of efficacy^35,36^. Our pipelines address the issues of metric summarization inconsistency and processing reproducibility across studies through the utilization of *PharmacoGx*, which efficiency standardizes the computation of drug sensitivity parameters for any pharmacogenomic dataset^12,30^.

The calculate From Raw function within *PharmacoGx* was used to compute the GRAY drug response data, while the computeSensitivity function was utilized to compute the UHNBreast drug response data^12^. The two functions reflect the data structure and formatting differences of the drug response data between the two breast cancer datasets.

### Incorporating published drug response data

The GRAY dataset includes published processed drug response data^14,17^. The published data was curated, annotated, and compiled into a PSet using *PharmacoGx*^12^. These metrics include growth inhibition (GI) and growth rate inhibition (GR): GI_50_, GR_50,_ GEC_50_, GR_max_, GR_inf_, h_GR_, and GR_AOC_. The sensitivity metrics can be defined as^14,17^:

GI_50_: the drug concentration for 50% inhibition of cell proliferation.

GR_50_: the drug concentration (c) to achieve *GR*(*c* = *GR50*) = *0*.*5*.

GEC_50_: the drug concentration for which 50% of maximal effect is observed.

GR_max_: the GR observed at the highest drug concentration.

GR_inf_: the effect of the utilization of an infinite drug concentration.

h_GR_: the fitted curve Hill coefficient.

GR_AOC_: the effect of a drug across AOC estimated concentrations.

### CWL pipeline execution steps

Each CWL pipeline within a workflow executes a custom R script with computational processing procedures for generating each PSet, which follow the same structure, regardless of the dataset being analyzed^11,12^. Each PSet that is generated begins with the execution of an R script that gathers curated identifiers for each cell line, tissue, and drug compound within each dataset. The curated identifiers are then used to collect cell line and drug metadata and generate a data array of the corresponding cell line and drug response experiment. The raw drug response data is then processed using *PharmacoGx*^12^, while the published drug response data is annotated and compiled. The pre-processed molecular profiles from each dataset are later organized into an ExpressionSet, which are data structures with processed data in the form of matrices with associated feature, phenotypic, and annotation data^37^. The last pipeline in our workflow compiles the curated unique identifiers, cell line and drug metadata, computed drug response data, published drug response data, and molecular profile ExpressionSets into a PSet through the PharmacoSet class in *PharmacoGx*^12^.

To execute a CWL workflow, cwltool must be run on the CWL and YAML files that are defined for a dataset workflow, where the –-provenance flag generates a Research Object^11^:

cwltool –-provenance /outputdir getUHN2017_Workflow.cwl getUHN2017_Workflow.yml

### Biomarker discovery

We utilized the GRAY 2017 and UHNBreast 2019 PSets to identify an association between ERBB2 expression and lapatinib drug response across cell lines^14–17,19^. We identified 39 and 50 cell lines from the GRAY and UHNBreast PSet’s, respectively, that possessed both ERBB2 gene expression and drug response data (AAC) for lapatinib. With AAC being one of the most commonly used drug sensitivity metrics, we utilized the gene expression and computed AAC data within the GRAY and UHNBreast PSets to assess this gene-drug association (expression-based biomarker) through calculating the concordance index and p-value^15,30,31^.

Our code for this analysis utilizes the summarizeSensitivityProfiles and summarizeMolecularProfiles functions in *PharmacoGx* to extract lapatinib response and ERBB2 expression data from the GRAY 2017 and UHNBreast 2019 PSets^12^. This response and expression data was subsequently used to compute the concordance index and p-value between them, for both PSets, using the concordance index function within the *survcomp* R package^38^.

## Data Availability

The GRAY and UHNBreast PSets generated through our CWL workflows can be found on Harvard Dataverse at 10.7910/DVN/BXIY5W ^28^, while the raw pharmacological and molecular data used in this manuscript for each respective study can be found on our Code Ocean capsule at 10.24433/CO.7378111.v3 ^29^. The GRAY RNA-seq, CNV, and 2017 drug response data used in this manuscript is available on Synapse (https://www.synapse.org/#!Synapse:syn2346643/wiki/62255). The GRAY processed methylation data is located on the NCBI Gene Expression Omnibus (GSE42944), while the mRNA (U133A and Exon 1.0 ST array) data is available from ArrayExpress (E-TABM-157 and E-MTAB-181). The UHNBreast RNA-seq data can be found on the NCBI Gene Expression Omnibus (GSE73526), while the remaining molecular profile data can be found at http://neellab.github.io/bfg/. The UHNBreast 2017 drug response data is available from PharmacoGx, while the 2019 data is available on our Code Ocean capsule.

## Code Availability

Our CWL workflows, a tutorial describing the installation and execution procedures for our workflows, and the code for our biomarker discovery analysis can be found on our Code Ocean capsule (10.24433/CO.7378111.v3)^29^.
